# Real-world patient characteristics and clinical outcomes in patients with myelofibrosis in Japan

**DOI:** 10.1371/journal.pone.0348598

**Published:** 2026-05-08

**Authors:** Yusuke Yasutomi, Masashi Takano, Yi-Chen Chen, Chi-Chang Chen, Seok-Won Kim, Catherine McGuiness, Alex Slowley, Keita Kirito

**Affiliations:** 1 Medical Affairs, GSK, Tokyo, Japan; 2 Real World Data Analytics, GSK, Tokyo, Japan; 3 Value Evidence & Outcomes APAC, GSK, Singapore, Singapore; 4 Real World Evidence Solutions, IQVIA, Wayne, United States of America; 5 Real World Evidence Solutions, IQVIA, Tokyo, Japan; 6 Health Economics and Outcomes Research, Real World Evidence, IQVIA, Wayne, United States of America; 7 Global Value Evidence and Outcomes (VEO), GSK, London, United Kingdom; 8 Department of Hematology/Oncology, University of Yamanashi, Yamanashi, Japan‌‌; Stanford University, UNITED STATES OF AMERICA

## Abstract

Anemia is associated with increased morbidity, mortality, healthcare resource utilization, and costs in myelofibrosis. This longitudinal, retrospective, and descriptive cohort study used a Japanese health administrative database (Medical Data Vision) to examine treatment patterns, transfusion burden, healthcare resource utilization, and costs in patients with myelofibrosis and a subset treated with a Janus kinase inhibitor. Ruxolitinib (a Janus kinase inhibitor) was the first targeted therapy for myelofibrosis in Japan (2014). Patients were identified from April 1, 2015 to June 30, 2022. In the overall myelofibrosis cohort, myelofibrosis diagnosis was the index date; in the Janus kinase inhibitor-treated subgroup, the first date of Janus kinase inhibitor use was the index date. Of the 836 patients with myelofibrosis, median age: 73 years at or before index; female: 38.0%; median follow-up: 576 days; anemia: 59.9%; thrombocytopenia: 18.7%. At index, 37.9%, 6.6%, and 55.5% of patients were transfusion-dependent, -requiring, and -independent, respectively. Median overall survival from myelofibrosis diagnosis was 83.3 months from the index date. Of 281 patients who received a Janus kinase inhibitor, median age: 74 years at or before index; female: 39.5%; median follow-up: 592 days; anemia: 66.9%; thrombocytopenia: 22.8%. All patients who received a JAK inhibitor were treated with ruxolitinib; mean dose was 15.6 mg/day and median duration of treatment was 14 months. The proportion of patients who were transfusion-dependent, -requiring, and -independent in this subgroup was 47.0%, 9.3%, and 43.8%, respectively. Median overall survival from myelofibrosis diagnosis was 53.9 months from first Janus kinase inhibitor administration. This real-world study showed the clinical characteristics and outcomes of Japanese patients with myelofibrosis.

## Introduction

Myelofibrosis (MF) is a myeloproliferative neoplasm that presents with bone marrow fibrosis, splenomegaly, and cytopenias, including anemia [1]. The disease can profoundly affect quality of life (QoL) and survival in patients with MF by increasing morbidity and mortality, as well as increasing healthcare resource utilization (HCRU) and costs [1,2].

Primary MF is classified as a rare disease by Orphanet, with an estimated worldwide prevalence of 1–9 cases per 100,000 individuals [3]; however, data specific for estimates in Japan are limited. A 17-year nationwide survey of Japanese patients with primary MF conducted between 1999 and 2015 estimated that 300 patients are diagnosed with primary MF annually, with a 3-year overall survival (OS) of 59% [4].

Anemia is the hallmark of MF, affecting about one-third of patients at diagnosis of primary MF [5]. Almost 50% of patients with MF who present with anemia will need red blood cell (RBC) transfusions within a year of diagnosis [5,6], and, eventually, most patients will become reliant on RBC transfusions, which are associated with low survival rates and diminished QoL [5–7]. The poor prognosis of patients with MF, as reflected by the severity of anemia, highlights a significant unmet clinical need.

Currently, the only curative treatment option for MF is hematopoietic stem cell transplantation (HSCT); however, use of HSCT is limited by its suitability for patients, which is determined by factors such as age, disease risk status, donor availability, performance status, comorbidities, and availability of nontransplant therapies [8]. Ruxolitinib, a Janus kinase (JAK) inhibitor, was approved as the first targeted therapy in Japan for MF based on the COMFORT trials. In pooled analyses of the COMFORT-I and -II trials, Japanese patients treated with ruxolitinib had reduced splenomegaly and improved MF-related symptoms and OS [9]. Ruxolitinib was the only JAK inhibitor approved in Japan for the treatment of MF until the approval of momelotinib in June 2024 [10]; prior to the approval of ruxolitinib, few therapeutic options were available for patients who discontinued, experienced relapse, or were ineligible for or refractory to ruxolitinib. Reasons for ruxolitinib discontinuation may include adverse events, such as anemia and thrombocytopenia, which may lead to dose reduction, lack or loss of spleen response, or disease progression [11].

There is a paucity of real-world data of patients with MF following the approval of ruxolitinib [12]. This retrospective cohort study describes the demographic and clinical characteristics, treatment patterns, transfusion burden, survival, HCRU, and costs associated with MF in the MDV database, including those treated with a JAK inhibitor.

## Materials and methods

### Ethics

This study complied with all applicable laws concerning patient privacy. No direct contact with patients or primary collection of individual patient data was involved. Data were accessed on November 15, 2023. All data were deidentified and the results of the study were aggregated and provided in tabular form, ensuring that patient identification was omitted. Therefore, obtaining informed consent, ethics committee approval, or institutional review board approval was not required.

### Objectives

The primary objectives of this retrospective study were to describe the demographic and clinical characteristics of patients with MF and those treated with a JAK inhibitor (i.e., ruxolitinib); treatment patterns, adherence, discontinuation, and ruxolitinib dosage; transfusion status and time to transfusion dependence; and the annual incidence and prevalence of MF within the Japan Medical Data Vision (MDV) database. Secondary objectives included determining the clinical outcomes, HCRU and costs, and number of patients with MF who are transfusion dependent (TD), transfusion requiring (TR), and transfusion independent (TI). The exploratory objective was to replicate analyses in patients with MF and anemia.

### Study design and population

This study was a longitudinal, retrospective, observational cohort study in Japanese patients with MF identified in the MDV database, which is a health administrative database containing anonymous health information of 40 million patients from 469 facilities, representing 26.6% of acute hospitals in Japan as of August 2024. Patients with MF were identified after the launch of ruxolitinib in Japan between April 1, 2015 and June 30, 2022 (the selection window). Patients were indexed at the date of initial (earliest) MF diagnosis in the overall MF cohort or the date of first JAK inhibitor use in the ruxolitinib subgroup. As MDV diagnoses were reported by month, the index date was the first day of the specified month (S1 Fig).

Inclusion criteria included the following: adult patients with ≥2 records at least 1 month apart with a diagnosis code indicative of confirmed MF (ICD10: C94.4, D47.4) during the selection window of April 1, 2015 to June 30, 2022; and MDV data visibility ≥6 months prior to the diagnosis index date (the baseline or pre-index period) and following the diagnosis index date of ≥1 month (the follow-up or post-index period). Exclusion criteria included the following: patients with ≥1 claim with a diagnosis code indicative of MF prior to the identification period; ≥2 records at least 1 month apart with a diagnosis of polycythemia vera (PV) or ≥2 records at least 1 month apart with a diagnosis of essential thrombocythemia (ET) after the MF index date; and ≥2 claims at least 1 month apart with a diagnosis code indicative of multiple myeloma, malignant lymphoma and/or myelodysplastic syndrome in the 6-month period before the MF index date. Patients diagnosed with anemia before ruxolitinib start date were not excluded from this study.

### Outcome definitions

#### Anemia outcomes.

Anemia was defined as a hemoglobin value ≤10 g/dL (in patients with available hemoglobin data), an anemia diagnosis, or an anemia treatment of interest (erythropoietin or epoetin alfa, danazol [androgen], blood transfusion). The anemia-finding window (for anemia status) began 100 days before the MF index date and continued until the first anemia diagnosis or treatment. The MF index date was the date of initial (earliest) MF diagnosis and the anemia index date was defined as the date of first anemia diagnosis or treatment or at the MF index date if initial anemia diagnosis or treatment occurred within the 100 days before the MF index date.

#### Transfusion outcomes.

The transfusion-finding period was defined as the 180-day period following the anemia index date (inclusive) and was used to assess the transfusion status. TD was defined as requirement of 6 units of RBC transfusions within a 12-week period or 4 units within an 8-week period; TI was defined as patients with 0 RBC transfusions; and TR was defined as patients with more than 0 RBC transfusions but who did not meet the definition of TD; non-TI was defined as patients who met the definition of TD or TR.

#### Line of treatment (LOT).

LOT was defined as the first, second, or third regimens. The identification of LOT is detailed in S1 Table. Each LOT was presented as a binary indicator variable (n, %) for regimens by drug names (up to 20 regimens). Duration of LOT was presented as months (median, range).

#### Mortality.

Mortality was defined as inpatient death (reported as observed in the hospital file) and was used as a proxy measure for OS. This method has been determined to be a robust alternative to identify mortality in patients with cancer by previous studies [13]. In the overall MF cohort, OS was measured from the date of initial MF diagnosis; in the ruxolitinib subgroup, OS was measured from the date of first JAK inhibitor use.

#### Ruxolitinib adherence.

Ruxolitinib adherence was defined as the proportion of days covered (PDC) over the 12-month follow-up period. Patients were considered adherent if PDC was ≥80%.

#### Healthcare costs.

MF-related all-cause healthcare costs in the post-index period was divided into the following categories: overall (inpatient hospital + outpatient disease management + inpatient/outpatient prescription fee with drug costs + transfusion costs), inpatient hospital costs (inpatient costs with an intensive care unit [ICU] stay during the hospitalization, inpatient costs without all-cause ICU stay or emergency department [ED] visit during the hospitalization, all-cause inpatient ED visit, all-cause blood transfusion during hospitalization), outpatient disease management costs (outpatient consultation costs, laboratory testing costs, surgical procedure costs, diagnostic imaging costs, radiotherapy costs, rehabilitation costs, blood transfusion [outpatient visit] costs), inpatient/outpatient (prescription costs, blood transfusion costs, MF-related all-cause anti-cancer medication orders, all-cause non-anti-cancer medication orders), and all-cause transfusion procedure costs.

### Statistical analysis

Descriptive statistics were generated for all study measures, including means, 95% confidence intervals, standard deviations (SD), medians for continuous variables and frequencies and percentages for the categorical variables. Study measures were reported for the overall sample and the anemia subgroup. Time to TD and rate of TD from MF index within 0.5/1/2/3/4-year intervals were estimated using Kaplan-Meier methods. Time to inpatient death was estimated using the Kaplan-Meier method from the start of each LOT (LOT1, 2, and 3) to date of inpatient death. HCRU/costs were reported descriptively as per person per month (PPPM) values (as mean [SD] 2024 ¥Japanese Yen) to adjust for the variable length of the post-index period. The post-index period for each patient ended on the date of loss of visibility, the end of the study period, or the date of inpatient death, whichever date occurred first. All analyses were conducted using SAS^®^ Release 9.4 (SAS Institute Inc., Cary, NC).

## Results

### Patient demographics and clinical characteristics

**Fig 1:**
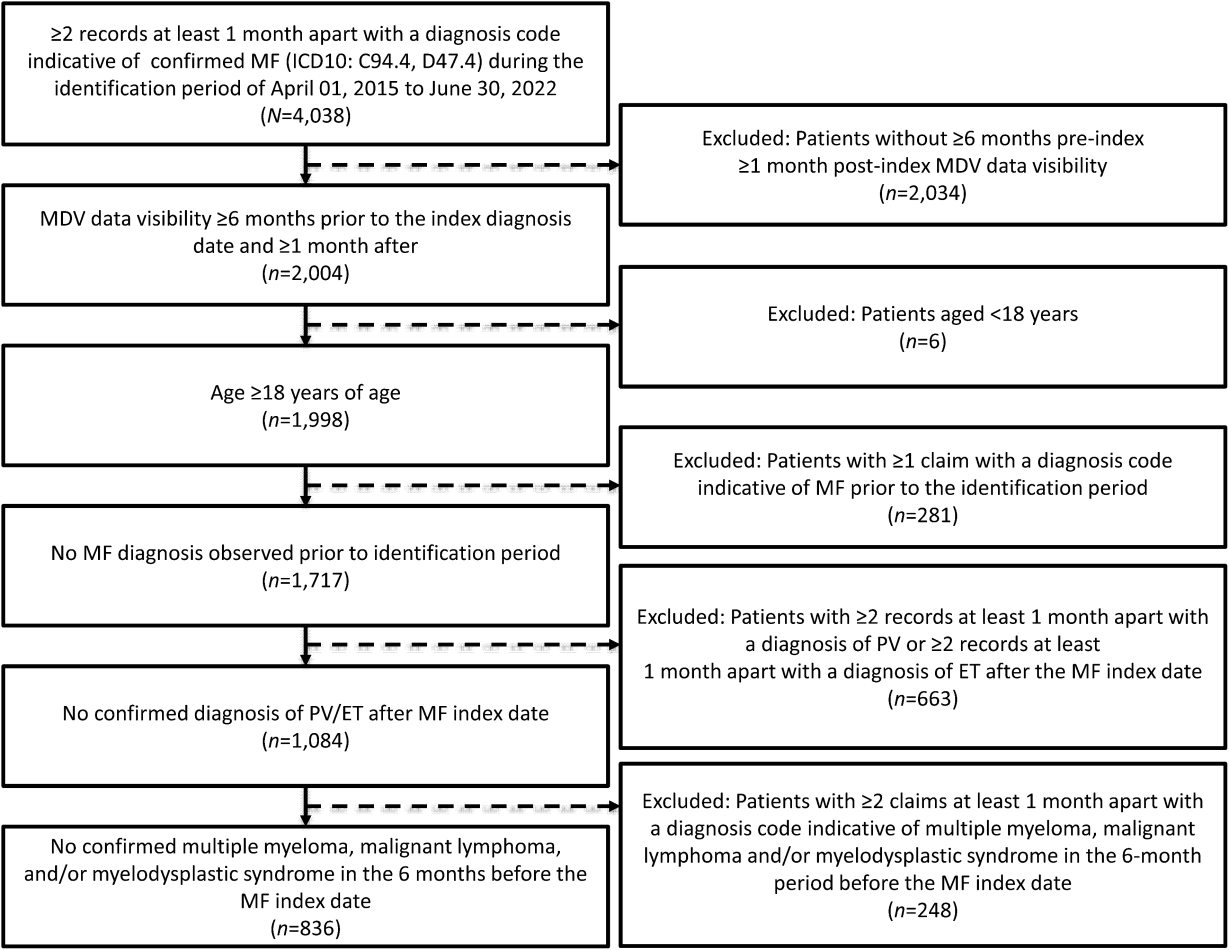
Patient disposition. *ET* essential thrombocythemia, *ICD* International Classification of Diseases, *MDV* Medical Data Vision, *MF* myelofibrosis, *PV* polycythemia vera.

A total of 836 patients in the MDV database met the selection criteria during the study period (Fig 1).

In the overall MF cohort, who were indexed at the date of initial (earliest) MF diagnosis, patients had a median (range) age of 73.0 (18.0–97.0) years (45.8% [383/836] were ≥75 years) and had a median (range) follow-up duration of over 1.5 years (576.0 [28.0–2928.0] days). Most patients were male (62.0% [518/836]) and the majority had primary MF (92.3% [772/836]). Anemia was observed in 59.9% (501/836) and thrombocytopenia in 18.7% (156/836); 20.1% (168/836) of patients had received transfusions at the date of the initial MF diagnosis (Table 1).

**Table 1:** Demographics and clinical characteristics.

	All patients with MF^a^	All patients with MF and ruxolitinib^b^
*N = 836*	*n = 281*
**Sex, n (%)**		
Male	518 (61.96)	170 (60.50)
Female	318 (38.04)	111 (39.50)
**Age (years)**		
Median	73.00	74.00
**Age group, n (%)**		
<65	193 (23.09)	58 (20.64)
65–74	260 (31.10)	85 (30.25)
≥75 years	383 (45.81)	138 (49.11)
**Duration of follow-up, days** ^**c**^		
Median	576.00	592.00
**Type of MF, n (%)**		
Primary MF	772 (92.34)	252 (89.68)
Secondary MF	64 (7.66)	29 (10.32)
**History of PV/ET in secondary MF**		
ET only	48 (75.00)	23 (79.31)
PT only	13 (20.31)	4 (13.79)
ET and PV	3 (4.69)	2 (6.90)
**MF-related comorbidities of interest, n (%)**		
Anemia	501 (59.93)	188 (66.90)
Thrombocytopenia	156 (18.66)	64 (22.78)
**Transfusion, n (%)**		
Yes	168 (20.10)	110 (39.15)

*ET* essential thrombocythemia, *MF* myelofibrosis, *PV* polycythemia vera, *SD* standard deviation. ^a^Index date: initial MF diagnosis. ^b^Index date: ruxolitinib initiation. ^c^Days from the index date to the end of the post-index period. The post-index period started on the index date and ended on the date of loss of data visibility, the end of the study period, or death during hospitalization, whichever occurred first

Of the 836 patients with MF, 281 (33.6%) received a JAK inhibitor; all 281 (281/281; 100%) received ruxolitinib (S2 Fig). The index date of this subgroup was the first date of JAK inhibitor use. Patients had a median (range) age of 74.0 (21.0–92.0) years (49.1% [138/281] were ≥75 years), majority were male (60.5% [170/281]), and the median (range) follow-up duration was over one year and a half (592.0 [3.0–2858.0] days). Most patients also had primary MF (89.7% [252/281]). Anemia was observed in 66.9% (188/281) and thrombocytopenia in 22.8% (64/281); transfusion was recorded for 39.2% (110/281) of patients (Table 1). All patients in this subgroup were treated with ruxolitinib (100.0% [281/281]), and 26.7% (75/281) of these patients had prior hydroxyurea treatment.

### Treatment patterns

In the overall MF cohort, 40.0% had at least one LOT, with the most common first LOT regimen being ruxolitinib alone (76.4% [255/334]).

In the ruxolitinib subgroup (n = 281), the most common first LOT was ruxolitinib alone (91.8% [258/281]), with the remaining patients receiving hydroxyurea and ruxolitinib combined (8.2% [23/281]) (S2 Table). A second LOT was observed in 10.0% (28/281) of patients, with the most common regimen being a combination of hydroxyurea and ruxolitinib (60.7% [17/28]) followed by hydroxyurea alone (39.3% [11/28]). A third LOT was observed in 0.7% (2/281) of patients, both of whom received a combination of hydroxyurea and ruxolitinib.

### Transfusion status and transfusion dependence

**Fig 2:**
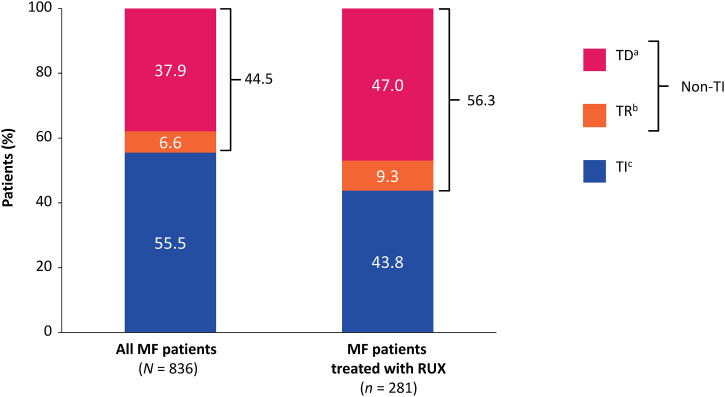
Transfusion status in patients with MF versus those treated with ruxolitinib. *MF* myelofibrosis, *RBC* red blood cells, *RUX* ruxolitinib, *TD* transfusion dependent, *TI* transfusion independent, *TR* transfusion requiring. ^a^Patients were classified as transfusion dependent if they received 6 units of RBCs within a span of 12 weeks during the post-anemia index period (i.e., after the anemia index date and within the transfusion-status finding period). Note: number of units transfused on each date were reported to the extent possible within the limitations of the database. If the number of units transfused could not be reliably identified, patients were assumed to have received 2 units of RBCs and a sensitivity analysis was performed, assuming more than and less than 2 units of RBC per visit (e.g., 1 unit and 3 units). ^b^Patients with anything more than 0 transfusions but who did not meet the definition of transfusion dependent. ^c^Patients with 0 transfusions during the post-anemia index period.

In the overall MF cohort, 37.9% (317/836) were TD, 6.6% (55/836) were TR, and 55.5% (464/836) were TI (Fig 2). Median (range) time to first transfusion (from MF index date) was 639 (0–2843) days; 52.4% (438/836) of patients received at least one transfusion. Rate of transfusion dependence (from MF index date) was 35.5% (*n* = 287) within 0.5 years, 37.0% (*n* = 297) within 1 year, 39.4% (*n* = 310) within 2 years, 40.8% (*n* = 315) within 3 years, 40.8% (*n* = 315) within 4 years.

In the ruxolitinib subgroup, 47.0% (132/281) were TD, 9.3% (26/281) were TR, and 43.8% (123/281) were TI (Fig 2). Median (range) time to first transfusion (from ruxolitinib initiation) was 158 (0–2633) days (Table 2); 61.9% (174/281) of patients received at least one transfusion. Rate of transfusion dependence (from initiation of ruxolitinib) was 44.3% (*n* = 80) within 0.5 years, 45.6% (*n* = 83) within 1 year, 48.8% (*n* = 89) within 2 years, 49.8% (*n* = 90) within 3 years, 49.8% (*n* = 90) within 4 years.

**Table 2:** Time to transfusion dependence and number of transfusions in patients treated with ruxolitinib.

	All MF patients treated with ruxolitinib	Anemia		Transfusion
Anemia	Non-anemia	TD
*N = 281*	*n = 230*	*n = 51*	*n = 132*
**Time to first transfusion after initiation of ruxolitinib**				
Median, days	158	56	NR	14
**Time from ruxolitinib initiation to TD** ^**a**^				
n (%)	240 (85.41)	189 (82.17)	51 (100.00)	91 (68.94)
Median, days	–	679	NR	35
**Number of transfusions, mean (SD)**				
Within 0.5 year	4.3 (6.6)	5.2 (7.0)	0.0 (0.0)	8.6 (7.4)
Within 1.0 year	7.3 (12.0)	8.9 (12.8)	0.0 (0.0)	14.4 (14.2)
Within 2.0 years	10.6 (18.1)	13.0 (19.2)	0.0 (0.0)	20.4 (21.8)
Within 3.0 years	12.7 (20.9)	15.5 (22.1)	0.0 (0.0)	24.0 (24.8)
Within 4.0 years	14.2 (23.5)	17.3 (24.9)	0.0 (0.0)	26.4 (28.4)

*MF* myelofibrosis, *NR* not reached, *SD* standard deviation, *TD* transfusion dependent. ^a^Estimated using the Kaplan-Meier method

### Ruxolitinib dosage, duration, and adherence

**Fig 3:**
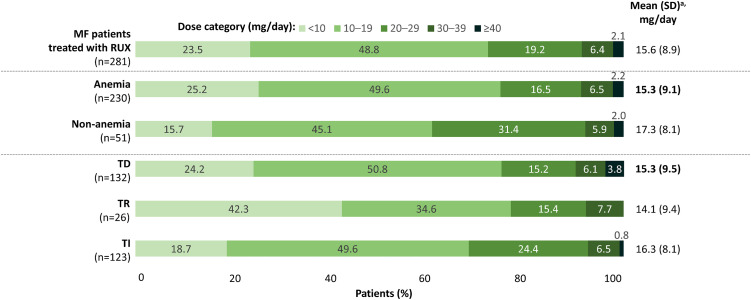
Ruxolitinib dosage and adherence. *MF* myelofibrosis, *RUX* ruxolitinib, *SD* standard deviation, *TD* transfusion dependent, *TI* transfusion independent. ^a^Index date: ruxolitinib initiation.

**Fig 4:**
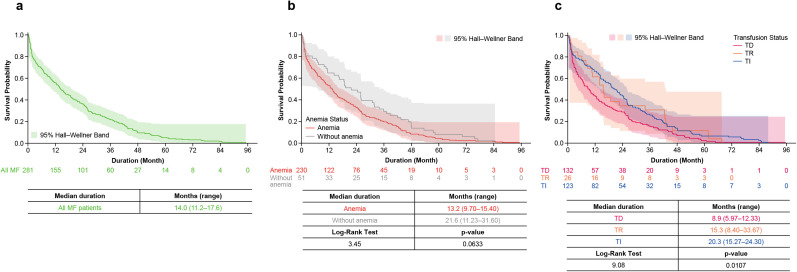
Duration of ruxolitinib treatment in the ruxolitinib subgroup. **(a)** All patients with MF; **(b)** Patients with MF by anemia status; **(c)** Patients with MF by transfusion status. *MF* myelofibrosis, *TD* transfusion dependent, *TI* transfusion independent, *TR* transfusion ‌‌requiring.

In the ruxolitinib subgroup, the mean (SD) dose was 15.6 (8.9) mg/day (Fig 3). Mean (SD) dose was 15.3 (9.5) mg/day in the TD group and 16.3 (8.1) mg/day in the TI group. Overall, the most common dose range was 10–19 mg/day (48.8%), followed by <10 mg/day (23.5%), and 20–29 mg/day (19.2%). This trend was also observed in the TD group. In the TI group, 10–19 mg/day was the most common dose range (49.6%), followed by 20–29 mg/day (24.4%), then <10 mg/day (18.7%). The proportion of patients who received ≥15 mg/day was 43.8% and 56.2% in those who received <15 mg/day in the follow-up period. The median duration (95% CI) of ruxolitinib treatment was 14.0 (11.2–17.6) months (Fig 4a). This was 13.2 (9.7–15.4) months in the anemia group and 21.6 (11.2–31.6) months in the non-anemia group (*p* = 0.0633; Fig 4b); in the TD group, it was 8.9 (6.0–12.3) months, 15.3 (8.4–33.7) months in the TR group, and 20.3 (15.3–24.3) months in the TI group (*p* = 0.0107; Fig 4c).

Overall, most patients (69.8% [132/189]) had a PDC score of ≥80% over 12 months of follow up and were considered adherent to ruxolitinib. The proportion of patients with a PDC score of ≥80% over 12 months of follow up in the anemia and non-anemia group was 67.1% (102/152) and 81.1% (30/37), respectively. For the TD, TR, and TI groups, the proportion of patients with a PDC score of ≥80% over 12 months of follow up was 60.8% (45/74), 63.2% (12/19), and 78.1% (75/96), respectively.

### Number of transfusions

**Fig 5:**
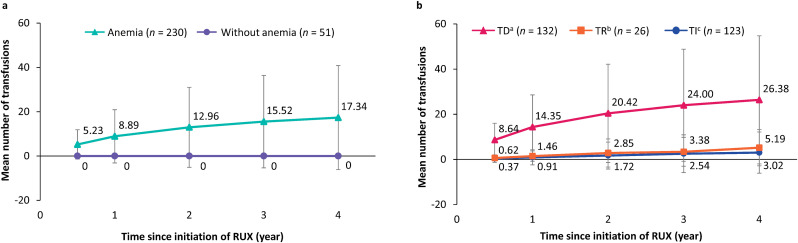
Mean (SD) number of transfusion procedures over time in patients treated with ruxolitinib. **(a)** With or without anemia; **(b)** By transfusion status. *RBC* red blood cell, *RUX* ruxolitinib, *SD* standard deviation, *TD* transfusion dependent, *TI* transfusion independent, *TR* transfusion requiring. ^a^Patients receiving ≥6 units of RBC within 12 weeks during the post-anemia index period (i.e., after the anemia index date and within the transfusion status–finding period); ^b^Patients receiving >0 transfusions but do not meet the definition of TD; ^c^Patients with 0 transfusions during the post-anemia index period.

In the overall MF cohort, the mean (SD) number of transfusion procedures from MF index date was 3.2 (5.9) within 0.5 years, 5.1 (9.8) within 1 year, 7.1 (14.3) within 2 years, 8.2 (16.1) within 3 years, and 9.0 (17.9) within 4 years. In the ruxolitinib subgroup, the mean (SD) number of transfusions was 4.3 (6.6) within 0.5 years, 7.3 (12.0) within 1 year, 10.6 (18.1) within 2 years, 12.7 (20.9) within 3 years, and 14.2 (23.5) within 4 years (Table 2). The mean (SD) number of transfusion procedures in patients with anemia was 5.2 (7.0) within 0.5 years, 8.9 (12.8) within 1 year, 13.0 (19.2) within 2 years, 15.5 (22.1) within 3 years, and 17.3 (24.9) within 4 years; patients without anemia had a mean (SD) of 0.0 (0.0) transfusions (Fig 5a). In the TD, TR, and TI groups, the mean (SD) number of transfusion procedures was 8.6 (7.4), 0.6 (0.8), and 0.4 (1.7) within 0.5 years, 14.4 (14.2), 1.5 (2.3), and 0.9 (3.3) within 1 year, 20.4 (21.8), 2.9 (6.2), and 1.7 (6.0) within 2 years, 24.0 (24.8), 3.4 (6.4), and 2.5 (8.3) within 3 years, and 26.4 (28.4), 5.2 (8.0), and 3.0 (9.1) within 4 years, respectively (Fig 5b).

### Clinical outcome

**Fig 6:**
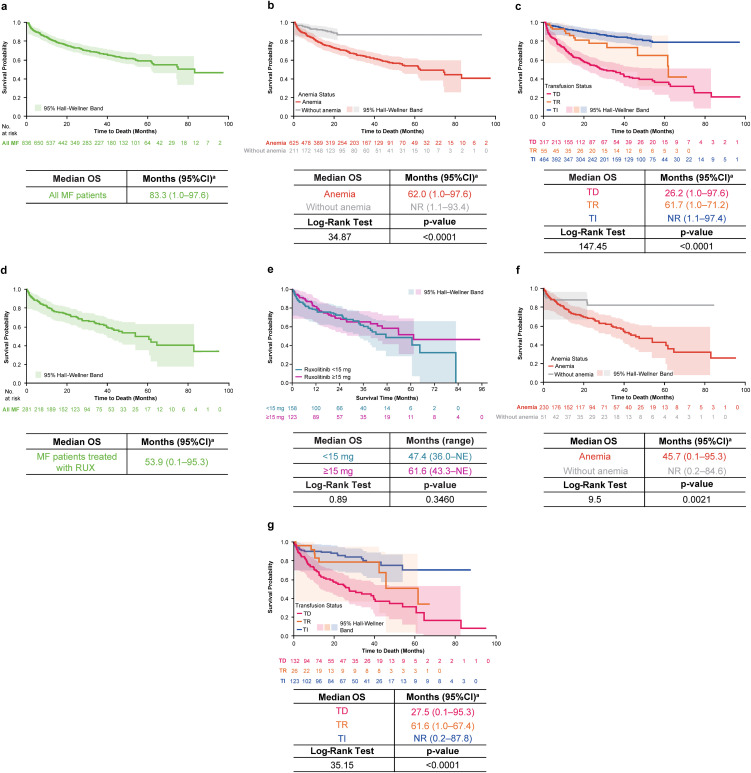
OS (inpatient death). **(a)** All patients with MF; **(b)** Patients with MF by anemia status; **(c)** Patients with MF by transfusion status; **(d)** Patients with MF and treated with ruxolitinib; **(e)** Patients with MF by ruxolitinib dosage (≥15 mg/day vs < 15 mg/day); **(f)** Patients with MF and treated with ruxolitinib by anemia status; **(g)** Patients with MF and treated with ruxolitinib by transfusion status. *MF* myelofibrosis, *NE* not estimable, *NR* not reached, *OS* overall survival, *RUX* ruxolitinib, *TD* transfusion dependent, *TI* transfusion independent, *TR* transfusion requiring. ^a^Death is proxied by the binary inpatient death variable (reported as observed in the hospital file). This study analyzed hospital mortality as a proxy measure for OS. Days between ruxolitinib initiation to the date of inpatient death (event of interest) among patients with ruxolitinib use was estimated using the Kaplan-Meier method.

In the overall MF cohort, inpatient death occurred in 27.8% (232/836) of patients. Unadjusted estimates of the mean time from initial MF diagnosis to date of inpatient death was 4.6 years; median (range) OS from MF diagnosis was 83.3 (1.0–97.6) months (Fig 6a). The median (range) OS for patients with anemia was 62.0 (1.0–97.6) months and was not reached for patients without anemia in the overall MF cohort (*p* < 0.0001; Fig 6b). The median (range) OS for patients who were TD was 26.2 (1.0–97.6) months, 61.7 (1.0–71.2) months for TR and not reached for those who were TI (*p* < 0.0001; Fig 6c)*.*

In the ruxolitinib subgroup, the mean estimated time from first JAK inhibitor use to date of inpatient death was 4.1 years; median (range) OS was 53.9 (0.1–95.3) months from first JAK inhibitor use (Fig 6d); for patients treated with ≥15 mg/day vs < 15 mg/day of ruxolitinib, median (95% CI) OS was 61.6 (43.3–NE) months vs 47.4 (36.0–NE) months (*p* = 0.3460; Fig 6e). The median (range) OS for patients in the ruxolitinib subgroup who had anemia was 45.7 (0.1–95.3) months and was not reached for those without anemia (*p* = 0.0021; Fig 6f)*.* The median (range) OS for patients who received ruxolitinib and were TD was 27.5 (0.1–95.3) months, 61.6 (1.0–67.4) months for TR, and not reached for those who were TI (*p* < 0.0001; Fig 6g).

### HCRU and costs

Mean (SD) all-cause PPPM estimates for patients treated with ruxolitinib were ¥733,800 (¥823,728) PPPM (S3 Table). The mean (SD) all-cause medication cost was ¥498,933 (¥537,268) PPPM for patients treated with ruxolitinib (S4 Table). Mean (SD) MF-related all-cause anti-cancer medication cost was ¥320,161 (¥263,174) PPPM, and the mean (SD) all-cause non-anti-cancer medication cost was ¥178,772 (¥465,458) PPPM.

In patients with anemia and no anemia, mean (SD) all-cause medication costs were ¥514,259 (¥567,555) PPPM and ¥429,817 (¥367,731) PPPM, respectively. For patients who were in the TD and TI groups, the mean (SD) all-cause medication costs were ¥634,044 (¥693,937) PPPM and ¥384,720 (¥304,190) PPPM, respectively.

Mean (SD) all-cause anti-cancer medication costs in patients with anemia and no anemia were ¥302,775 (¥238,565) PPPM and ¥398,568 (¥345,738) PPPM, respectively; mean (SD) all-cause non-anti-cancer medication costs were ¥211,484 (¥506,370) PPPM and ¥31,249 (¥108,460) PPPM. In patients who were TD, the mean (SD) all-cause anti-cancer medication costs were ¥296,336 (¥236,080) PPPM; in those who were TI, costs were ¥350,580 (¥291,440) PPPM. The mean (SD) all-cause non-anti-cancer medication costs were ¥337,708 (¥635,108) PPPM and ¥34,140 (¥95,315) PPPM in patients who were TD and TI, respectively.

For costs associated with blood transfusion, the mean (SD) all-cause outpatient blood transfusion cost was ¥35,028 (¥58,975) PPPM in patients treated with ruxolitinib (S4 Table). Mean (SD) all-cause outpatient blood transfusion costs in patients with anemia were ¥42,578 (¥62,698) PPPM and ¥980 (¥5,297) PPPM in patients without anemia. In patients who were TD, the mean (SD) all-cause outpatient blood transfusion costs were ¥68,585 (¥70,997) PPPM and ¥3,944 (¥10,061) PPPM in patients who were TI.

### Annual incidence and prevalence of MF

The MDV database census increased from 8,004,441 patients overall in 2015 to 12,474,824 patients in 2022. The incidence of MF was 0.44 annual cases per 100,000 people in 2015 and 1.32 per 100,000 people in 2020 and 2021. The prevalence of MF was 0.44 cases per 100,000 in 2015 and 5.32 per 100,000 people in 2022 (S3 Fig).

## Discussion

This real-world study used the MDV database, the largest electronic health record database in Japan, to identify and investigate the demographic and clinical characteristics, treatment patterns and trends, transfusion status, survival, HCRU, and costs in patients with MF after the launch of ruxolitinib in Japan. Eligible patients were identified between April 1, 2015 and June 30, 2022, and analyses were performed on the overall MF cohort as well as a subset of the cohort who received ruxolitinib, the only approved JAK inhibitor in Japan at the time of the study (momelotinib was approved in June 2024) [10]. The subset of ruxolitinib-treated patients was further stratified by anemia and transfusion status.

Using the MDV database, this study found an annual incidence of MF of 0.44 annual cases per 100,000 individuals in 2015 and 1.32 annual cases per 100,000 individuals in 2021. These results largely align with estimations from a Japanese research group on idiopathic hematopoietic disorders, which reported 0.3 cases of MF per 100,000 individuals in Japan [4], and other sources such as Orphanet, which estimated a worldwide prevalence of 1–9 cases per 100,000 individuals [3]. Although this study’s results support those reported by other publications, it is important to keep in mind that our study represents one sample of Japanese patients with MF between 2015 and 2022.

In this study, patients in the overall MF cohort had a median age of 73 years, 62% were male, and most patients were categorized as having primary MF (92.3%). While data related to MF in Japan are limited, the demographics of this study are similar to that of a post-marketing survey for ruxolitinib in MF, which reported a median age of 70 years, of whom 55% were males [14]. The proportion of patients with primary MF versus secondary MF in this study is higher than myelofibrosis clinical trials, such as COMFORT-I and COMFORT-II [15,16], which may be due to the exclusion of patients who were diagnosed with PV/ET after MF diagnosis for this study. This exclusion criterion was applied because it was not possible to determine whether patients had MF or PV/ET for those who were diagnosed with PV/ET after being diagnosed with MF.

Of the patients treated with ruxolitinib, almost half were TD and more than half of patients were non-TI. The median duration of ruxolitinib treatment was 14.0 months, which may indicate a slightly longer time on therapy for ruxolitinib compared with other agents, such as hydroxyurea [17]. Although the demographics differ slightly, the findings here align with a study reported by Passamonti, et al. (2022), which found a median duration of 13.1 months on ruxolitinib [18]. Although patients without anemia had a longer duration of ruxolitinib treatment, the difference was not statistically significant when compared with those who had anemia (*p* = 0.0633). However, the numerically longer duration of ruxolitinib in patients without anemia may be reflective of the higher proportion of patients adhering to the treatment compared with those who had anemia. When focusing on transfusion status, the duration of treatment was longer in patients who were TI than TD, demonstrating that reduced anemia rates and transfusion burden may help patients remain on treatment longer. However, it is important to acknowledge that another factor that affects ruxolitinib treatment duration is disease progression, which was not explored in this study; further, other baseline disease or patient characteristics that could have impacted ruxolitinib treatment duration—such as age and molecular risk factors—were not investigated in this study.

Most patients with anemia and those who were TD were treated with <20 mg/day of ruxolitinib, which is less than the daily doses received by those without anemia or were TI, suggesting that those with anemia or who were TD were less able to tolerate ruxolitinib treatment. However, it is important to note that, overall, patients received a mean dose of 15.6 mg/day, which is lower than the recommended dose in Japan (17.7 mg/day) [14], likely due to concerns around anemia. However, the REALISE phase 2 study found that it is unnecessary to delay or withhold ruxolitinib due to co-existent or treatment-emergent anemia [19]. Additionally, real-world studies, including interim results from an Italian observational study [20], have reported that a reduced dose of ruxolitinib is associated with less favorable outcomes, including OS [12]. However, this study found that there was no statistical difference in OS between patients who received <15 mg/day and those who received ≥15 mg/day (47.4 and 61.6 months, respectively; *p* = 0.3460), showing that further research is needed to clarify the relationship between ruxolitinib dosage and OS outcomes. Notably, ruxolitinib dosing was at the treating physician’s discretion, and reasons for dose adjustment were not recorded, limiting the interpretation of these findings. Furthermore, this study did not adjust for background characteristics between the two groups, which could have impacted OS outcomes.

In the overall MF cohort, which included patients treated with ruxolitinib, the median OS (assessed using inpatient death as a proxy) was 83.3 months from the index date at MF diagnosis. This study also showed longer OS in patients without anemia than those with anemia and in patients who were TI than those who were TD. This is supported by findings from the COMFORT studies, which found that the presence of anemia at baseline was associated with worse survival [21], and another study that found patients who received transfusions had worse survival compared with those who did not [22]. For Japanese patients, a nationwide survey study involving approximately 500 hematology departments and 780 patients with newly diagnosed primary MF found that a requirement for transfusions was one of the most significant predictors of shorter survival [4]. The mortality associated with anemia and TD in patients with MF highlights the need for improved treatments to manage and/or prevent anemia and TD in patients with MF.

Among patients treated with ruxolitinib, the median OS was 53.9 months from the index date at first ruxolitinib use, which is different to the overall MF cohort as the OS estimates are based on different index dates and reflect differences in patient characteristics. In Japan, patients are only eligible for ruxolitinib if they are at an intermediate-II risk or higher according to the Dynamic International Prognostic Scoring System for primary MF. This means there are likely fewer patients with poor prognosis at baseline in the overall MF group compared with patients treated with ruxolitinib. These factors may explain the longer OS rates observed in the overall MF cohort versus that of patients treated with ruxolitinib. In the ruxolitinib subgroup, patients without anemia or who were TI had longer OS than those with anemia or who were TD, respectively.

In patients treated with ruxolitinib, those with anemia incurred higher total costs than those without anemia; patients who were TD had higher total costs than those who were TI. Furthermore, this study found that anti-cancer medication costs were higher in patients without anemia and in those who were TI than those with anemia and who were TD. On the other hand, non-anti-cancer medication costs in patients with anemia were higher than in those without anemia. This suggests that patients without anemia and those who were TI were able to stay on JAK inhibitor treatment for longer, resulting in higher anti-cancer medication costs.

This study has some limitations. Firstly, the MDV data were collected primarily for patient care and not for research purposes. As such, the utility of leveraging the MDV database for research is limited by the completeness and accuracy of the underlying data. Data that are not recorded, miscoded, or fail to accurately describe clinical diagnoses or treatment have the potential to introduce bias, including the incidence and prevalence of MF, as it is limited to patients in the database; however, the analysis was performed with this in mind to minimize such potential biases. In addition, the MDV data were sourced from hospital systems; therefore, services, treatments, laboratory results, and outpatient deaths may not be captured if they are billed outside of the data collection system, which may under or overestimate all-cause HCRU, costs, and clinical outcomes. Another limitation of this study is its observational design, which may lead to selection and attrition bias, as well as the lack of a control group. Any observed or described differences found between groups could be due to unbalanced baseline characteristics, such as age, disease-related anemia, and cytopenic phenotype, particularly as established prognostic scores like the Dynamic International Prognostic Scoring System and molecular risk factors were not captured in the database. Therefore, care should be taken in the interpretation of results, especially for groups that had relatively small patient numbers, specifically, the subset of patients without anemia receiving ruxolitinib treatment. Lastly, in the ruxolitinib subgroup, survival was measured from treatment initiation, while anemia and transfusion status may evolve over time; the potential for immortal time bias should considered when interpreting these data.

Strengths of the study include the fact that the MDV database is a largescale database that covers approximately 26.6% of acute hospitals in Japan, providing the possibility of analyzing data in rare diseases, particularly in the elderly population [23]. Additionally, the database is updated monthly, providing an up-to-date source of real-world data, and has been used as a data source in many studies across therapy areas [23].

This is the first study of Japanese patients with MF using the MDV database to investigate real-world patient characteristics, clinical outcomes and economic impact in Japan. This study highlighted the burden of anemia and transfusion in patients with MF, along with shorter survival in patients with anemia in the overall cohort and the JAK inhibitor subgroup. Furthermore, non-anti-cancer medication costs in patients without anemia were higher than in those with anemia. These findings highlight the need for new modalities of treatment for patients with MF to address anemia and associated transfusion requirements for patients in Japan.

## Supporting information

S1 File Supplementary material_Plain language summary. (DOCX)

S1 Table Identification of lines of therapy (LOT). (DOCX)

S2 Table Treatment regimens. (DOCX)

S3 Table Overall costs in all patients with MF and patients treated with JAK inhibitor. (DOCX)

S4 Table Prescription and transfusion costs associated with MF in all patients and patients treated with JAK inhibitor. (DOCX)

S1 Fig Study design. (DOCX)

S2 Fig Populations of interest. (DOCX)

S3 Fig Incidence^a^ and prevalence^b^ of patients with MF in MDV. (DOCX)
