# Supplementary material for: Real-world patient characteristics and clinical outcomes in patients with myelofibrosis in Japan
Source: PLoS One. 2026 May 8;21(5):e0348598. doi: 10.1371/journal.pone.0348598 (PMC13155682; doi:10.1371/journal.pone.0348598)
Supplement: S1 File — (DOCX) [file pone.0348598.s001.docx]

**S1** **Supplementary material. Plain language summary.**

**Why was this study/research done?**

- Myelofibrosis is a rare type of bone marrow cancer that can cause low red blood cell levels (anemia) among other symptoms, leading to weakness and tiredness that may need to be treated with blood transfusions
- A type of medication used to treat myelofibrosis is called Janus kinase (JAK) inhibitors, which reduce the number of cancer cells
  - While JAK inhibitors are used to treat many symptoms of myelofibrosis, they can also cause or worsen anemia in people with myelofibrosis
- Because myelofibrosis is a rare disease, there is little information on the clinical outcomes and economic impact in people with myelofibrosis, especially in Japan

**What did the researchers do?**

- Using a large medical database of Japanese people, we searched for information about those with myelofibrosis
- We looked at the medical history of these people between 2015 and 2022
- We wanted to determine:

1. The characteristics of people who have myelofibrosis, such as age and sex
2. How myelofibrosis is treated
3. The survival outcomes of patients with myelofibrosis
4. The costs to patients with myelofibrosis
5. How common myelofibrosis is in Japan

**What did the researchers find?**

- We found that people with myelofibrosis were typically male and in their 70s, with most having anemia
- Ruxolitinib is a JAK inhibitor that is the most common treatment for MF
- Nearly half of the patients were dependent on blood transfusions to treat their anemia, regardless of the treatment received
- We confirmed that people with myelofibrosis who did not have anemia survived longer than those with anemia
- We also confirmed that people who did not depend on blood transfusions survived longer than those who needed blood transfusions
- Non-anti-cancer medication costs in patients without anemia were higher than those with anemia

**What do these findings mean for patient care?**

- These findings show the need for therapies that will help improve management of myelofibrosis and its associated anemia for patients in Japan
