# Supplementary material for: Real-world patient characteristics and clinical outcomes in patients with myelofibrosis in Japan
Source: PLoS One. 2026 May 8;21(5):e0348598. doi: 10.1371/journal.pone.0348598 (PMC13155682; doi:10.1371/journal.pone.0348598)
Supplement: S1 Table — (DOCX) [file pone.0348598.s002.docx]

**S1 Table. Identification of lines of therapy (LOT).**

| **Identification step** | **Identification rules** |
| --- | --- |
| 1. Identify the first observed LOT | - The first observed claim for an MF treatment of interest (e.g., ruxolitinib, hydroxyurea) or allogenic SCT during the whole study period will be used to identify the first LOT |
| 1. Identify all the MF treatments of interest in the first LOT | - All treatments of interest observed within 28 days after the first claim for the first MF treatment will be included in the first LOT - Among patients with SCT, conditioning therapy prior to SCT will be included in the SCT LOT |
| 1. Identify LOT advancements | The LOT will be advanced if any of the following occur:   - A new MF treatment is added after the initial 28 days after the start of the LOT - Discontinuation of all agents in the regimen (defined by a gap in coverage of >60 days for all of the MF treatments in the regimen)   - A discontinued regimen restarted at a later date will be counted as a new LOT if there is ≥1 other regimens administered in between   - A discontinued regimen restarted at a later date with no other regimens administered in between will be considered the same LOT (will not advance the line) - Each SCT will be considered a new LOT comprised of conditioning therapy and the SCT procedure itself. Any chemotherapy agents appearing in the 8-day conditioning period preceding the SCT date of service will be included in the SCT regimen and will initiate the SCT LOT   - SCT LOTs begin on the first day of conditioning (or SCT procedure date if no conditioning) and end on the SCT procedure date. |
| 1. Determine the end of the LOT | - The LOT will end on the earliest (first) date that either of the following occur: 1) the last day of treatment for all the MF - treatments in the regimen; or 2) the date the LOT is advanced due to addition of a new MF treatment - The last day of treatment will be the date of the last administration claim (for injectable medications) or the date of the last prescription claim plus the days’ supply (for oral medications) |

MF, myelofibrosis; LOT, line of treatment; SCT, stem cell transplant
