# Supplementary material for: Real-world patient characteristics and clinical outcomes in patients with myelofibrosis in Japan
Source: PLoS One. 2026 May 8;21(5):e0348598. doi: 10.1371/journal.pone.0348598 (PMC13155682; doi:10.1371/journal.pone.0348598)
Supplement: S2 Table — (DOCX) [file pone.0348598.s003.docx]

**S2 Table. Treatment regimens.**

|  | **All MF patients treated with ruxolitinib^a^** |
| --- | --- |
|  | N=281 |
| **First LOT regimens, n (%)^b^** | 281 (100.00%) |
| Hydroxyurea + ruxolitinib | 23 (8.19%) |
| Ruxolitinib | 258 (91.81%) |
| **Second LOT regimens, n (%)^b^** | 28 (9.96%) |
| Hydroxyurea | 11 (39.29%) |
| Hydroxyurea + ruxolitinib | 17 (60.71%) |
| **Third LOT regimens, n (%)^b^** | 2 (0.71%) |
| Hydroxyurea + ruxolitinib | 2 (100.00%) |

^a^Index date: JAK inhibitor initiation. ^b^By drug name. JAK, Janus kinase; LOT, line of treatment
