# Supplementary material for: Real-world patient characteristics and clinical outcomes in patients with myelofibrosis in Japan
Source: PLoS One. 2026 May 8;21(5):e0348598. doi: 10.1371/journal.pone.0348598 (PMC13155682; doi:10.1371/journal.pone.0348598)
Supplement: S3 Table — (DOCX) [file pone.0348598.s004.docx]

**S3 Table. Overall costs in all patients with MF and patients treated with JAK inhibitor.**

| **Overall (inpatient hospital + outpatient disease management + inpatient/outpatient prescription fee with drug costs)** | **All MF patients** | **Anemia** | | **Transfusion status** | |
| --- | --- | --- | --- | --- | --- |
|  |  | Anemia | Non-anemia | TD | TI |
|  | N=836 | n=625 | n=211 | n=317 | n=464 |
| **All-cause costs, PPPM** | | | | | |
| Mean | ¥508,751.06 | ¥608,579.77 | ¥213,049.90 | ¥947,683.63 | ¥228,042.63 |
| SD | ¥1,064,179.32 | ¥1,199,378.18 | ¥333,177.57 | ¥1,585,861.49 | ¥315,506.61 |
|  | **All MF patients treated with JAK inhibitor** |  | | | |
|  | N=281 | n=230 | n=51 | n=132 | n=123 |
| **All-cause costs, PPPM** | | | | | |
| Mean | ¥733,800.46 | ¥775,118.39 | ¥547,464.70 | ¥994,983.35 | ¥500,347.82 |
| SD | ¥823,727.54 | ¥878,827.50 | ¥468,162.46 | ¥1,062,809.71 | ¥412,395.41 |

JAK, Janus kinase; MF, myelofibrosis; PPPM, per patient per month; SD, standard deviation; TD, transfusion dependent; TI, transfusion independent
