# Supplementary material for: Real-world patient characteristics and clinical outcomes in patients with myelofibrosis in Japan
Source: PLoS One. 2026 May 8;21(5):e0348598. doi: 10.1371/journal.pone.0348598 (PMC13155682; doi:10.1371/journal.pone.0348598)
Supplement: S4 Table — (DOCX) [file pone.0348598.s005.docx]

**S4 Table. Prescription and transfusion costs associated with MF in all patients and patients treated with JAK inhibitor**

| **Inpatient/outpatient:  Prescription costs, blood transfusion costs** | **All MF patients** | **Anemia** | | **Transfusion status** | |
| --- | --- | --- | --- | --- | --- |
|  |  | Anemia | Non-anemia | TD | TI |
|  | N=836 | n=625 | n=211 | n=317 | n=464 |
| **Prescription** | | | | | |
| **All-cause medication order costs, PPPM^a^** | | | | | |
| Mean | ¥282,007.50 | ¥341,702.16 | ¥105,186.83 | ¥538,714.81 | ¥119,968.95 |
| SD | ¥822,764.32 | ¥937,771.33 | ¥191,070.29 | ¥1,273,377.20 | ¥190,216.21 |
| **All-cause anti-cancer medication order costs, PPPM^a^** | | | | | |
| Mean | ¥116,358.38 | ¥124,873.54 | ¥91,135.77 | ¥146,467.16 | ¥95,199.61 |
| SD | ¥188,681.46 | ¥191,131.12 | ¥179,305.78 | ¥203,599.46 | ¥175,847.11 |
| **All-cause non-anti-cancer medication order costs, PPPM^a^** | | | | | |
| Mean | ¥165,649.12 | ¥216,828.62 | ¥14,051.06 | ¥392,247.66 | ¥24,769.35 |
| SD | ¥780,037.46 | ¥896,210.25 | ¥42,772.42 | ¥1,231,981.75 | ¥61,137.62 |
| **Blood transfusion** | | | | | |
| **All-cause outpatient blood transfusion costs, PPPM^a^** | | | | | |
| Mean | ¥26,335.53 | ¥35,178.46 | ¥142.01 | ¥65,271.11 | ¥1,889.03 |
| SD | ¥50,967.96 | ¥56,259.92 | ¥1,371.08 | ¥65,737.63 | ¥6,608.67 |
|  | **All MF patients  treated with JAK inhibitor** |  | | | |
|  | N=281 | n=230 | n=51 | n=132 | n=123 |
| **Prescription** | | | | | |
| **All-cause medication order costs, PPPM^a^** | | | | | |
| Mean | ¥498,932.81 | ¥514,258.55 | ¥429,816.74 | ¥634,043.92 | ¥384,720.33 |
| SD | ¥537,268.28 | ¥567,554.57 | ¥367,730.87 | ¥693,936.81 | ¥304,190.10 |
| **All-cause anti-cancer medication order costs, PPPM^a^** | | | | | |
| Mean | ¥320,160.77 | ¥302,774.86 | ¥398,567.80 | ¥296,336.31 | ¥350,580.46 |
| SD | ¥263,174.11 | ¥238,565.25 | ¥345,738.49 | ¥236,080.12 | ¥291,440.41 |
| **All-cause non-anti-cancer medication order costs, PPPM^a^** | | | | | |
| Mean | ¥178,772.04 | ¥211,483.69 | ¥31,248.95 | ¥337,707.61 | ¥34,139.87 |
| SD | ¥465,457.88 | ¥506,370.29 | ¥108,459.54 | ¥635,108.11 | ¥95,314.93 |
| **Blood transfusion** | | | | | |
| **All-cause outpatient blood transfusion costs, PPPM^a^** | | | | | |
| Mean | ¥35,028.03 | ¥42,577.82 | ¥979.96 | ¥68,584.50 | ¥3,944.06 |
| SD | ¥58,974.47 | ¥62,697.77 | ¥5,296.53 | ¥70,997.31 | ¥10,060.70 |

^a^Measured among all patients
JAK, Janus kinase; MF, myelofibrosis; PPPM, per patient per month; SD, standard deviation; TD, transfusion dependent; TI, transfusion independent
