# Supplementary material for: Real-world patient characteristics and clinical outcomes in patients with myelofibrosis in Japan
Source: PLoS One. 2026 May 8;21(5):e0348598. doi: 10.1371/journal.pone.0348598 (PMC13155682; doi:10.1371/journal.pone.0348598)
Supplement: S1 Fig — (DOCX) [file pone.0348598.s006.docx]

**S1 Fig. Study design.**

1 Apr 2008

1 Apr 2015

30 Jun 2022

30 Jun 2023

**Index event of interest:
Earliest observed MF diagnosis**

(re-index to ruxolitinib start)

**Follow-up period (≥1 month):**

- MF incidence/prevalence
- Anemia treatment
- LoT1/LoT2/LoT3 treatment patterns (including adherence/discontinuation)
- TI, non-TI (TR and TD), TD, TR
- Clinical outcomes (DoT, OS)
- HCRU and costs
- Rate/timing of progression to AML

**Pre-index period (≥6 months and at ruxolitinib initiation):**

- Demographic characteristics
- Clinical characteristics
- Prior diagnosis/comorbidities of interest
- Anemia treatment

**Index period**

**Post-index/follow-up
period**

**Pre-index/baseline
period**

A

B

100 days

**MF index**

Transfusion status-finding period

Anemia-finding period

180 days

**Anemia index**

Follow-up

**Note: if anemia was identified before MF index, anemia index is MF index**

DOT, duration of treatment; LOT, line of treatment; MF, myelofibrosis; OS, overall survival; TD, transfusion dependent; TI, transfusion independent; TR, transfusion requiring
