# Supplementary material for: Real-world patient characteristics and clinical outcomes in patients with myelofibrosis in Japan
Source: PLoS One. 2026 May 8;21(5):e0348598. doi: 10.1371/journal.pone.0348598 (PMC13155682; doi:10.1371/journal.pone.0348598)
Supplement: S2 Fig — (DOCX) [file pone.0348598.s007.docx]

**S2 Fig. Populations of interest.**


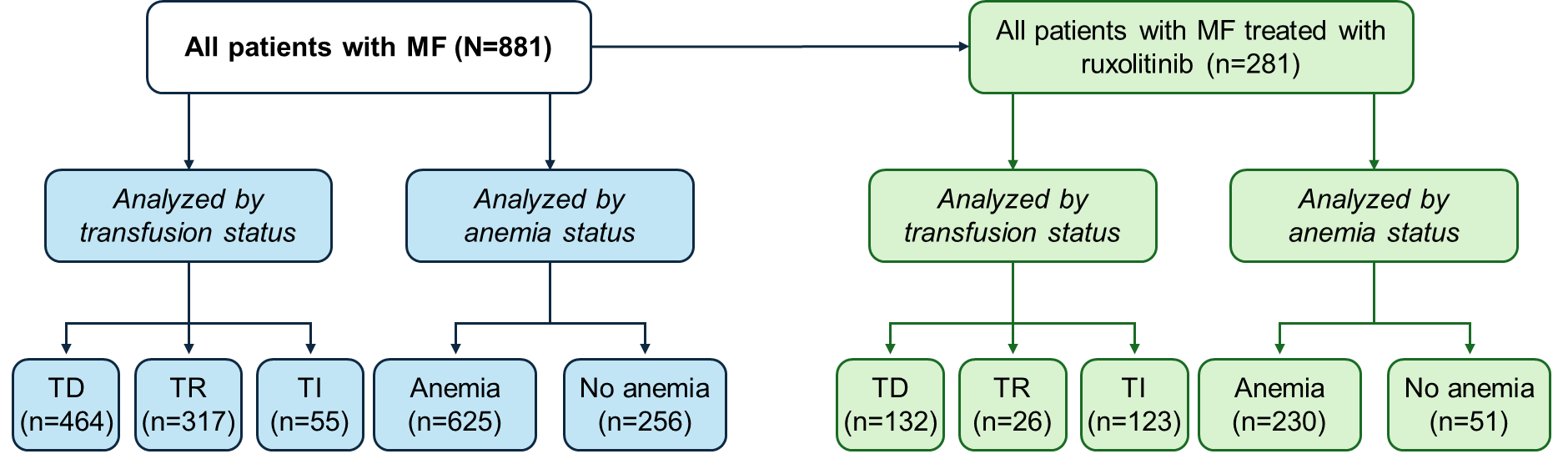


MF, myelofibrosis; TD, transfusion dependent; TI, transfusion independent; TR, transfusion requiring
