# Supplementary material for: Real-world patient characteristics and clinical outcomes in patients with myelofibrosis in Japan
Source: PLoS One. 2026 May 8;21(5):e0348598. doi: 10.1371/journal.pone.0348598 (PMC13155682; doi:10.1371/journal.pone.0348598)
Supplement: S3 Fig — (DOCX) [file pone.0348598.s008.docx]

**S3 Fig. Incidence^a^ and prevalence^b^ of patients with MF in MDV.**

^a^New MF cases: Defined as number of MF patients indexed to the given calendar year without an MF diagnosis in all available history in the MF data extract IQVIA received. ^b^Prevalent MF cases: Defined as cumulative number of MF patients with MF diagnosis from history to the given calendar year (and still alive in the given year). MDV, Medical Data Vision; MF, myelofibrosis
